# Nomograms Involving HER2 for Predicting Lymph Node Metastasis in Early Gastric Cancer

**DOI:** 10.3389/fcell.2021.781824

**Published:** 2021-12-24

**Authors:** Yu Mei, Shuo Wang, Tienan Feng, Min Yan, Fei Yuan, Zhenggang Zhu, Tian Li, Zhenglun Zhu

**Affiliations:** ^1^ Department of General Surgery, Gastrointestinal Surgery, Shanghai Key Laboratory of Gastric Neoplasms, Shanghai Institute of Digestive Surgery, Ruijin Hospital, Shanghai Jiao Tong University School of Medicine, Shanghai, China; ^2^ State Key Laboratory of Medical Genomics, Shanghai Institute of Hematology, Ruijin Hospital, Shanghai Jiao Tong University School of Medicine, Shanghai, China; ^3^ Clinical Research Institute, Shanghai Jiao Tong University School of Medicine, Shanghai, China; ^4^ Department of Pathology, Ruijin Hospital, Shanghai Jiao Tong University School of Medicine, Shanghai, China; ^5^ School of Basic Medicine, Fourth Military Medical University, Xi’an, China

**Keywords:** early gastric cancer, lymph node metastasis, nomogram, HER2, prediction model

## Abstract

**Objective:** We aimed to establish a nomogram for predicting lymph node metastasis in early gastric cancer (EGC) involving human epidermal growth factor receptor 2 (HER2).

**Methods:** We collected clinicopathological data of patients with EGC who underwent radical gastrectomy and D2 lymphadenectomy at Ruijin Hospital, Shanghai Jiao Tong University School of Medicine between January 2012 and August 2018. Univariate and multivariate logistic regression analysis were used to examine the relationship between lymph node metastasis and clinicopathological features. A nomogram was constructed based on a multivariate prediction model. Internal validation from the training set was performed using receiver operating characteristic (ROC) and calibration plots to evaluate discrimination and calibration, respectively. External validation from the validation set was utilized to examine the external validity of the prediction model using the ROC plot. A decision curve analysis was used to evaluate the benefit of the treatment.

**Results:** Among 1,212 patients with EGC, 210 (17.32%) presented with lymph node metastasis. Multivariable analysis showed that age, tumor size, submucosal invasion, histological subtype, and HER2 positivity were independent risk factors for lymph node metastasis in EGC. The area under the ROC curve of the model was 0.760 (95% CI: 0.719–0.800) in the training set (*n* = 794) and 0.771 (95% CI: 0.714–0.828) in the validation set (*n* = 418). A predictive nomogram was constructed based on a multivariable prediction model. The decision curve showed that using the prediction model to guide treatment had a higher net benefit than using endoscopic submucosal dissection (ESD) absolute criteria over a range of threshold probabilities.

**Conclusion:** A clinical prediction model and an effective nomogram with an integrated HER2 status were used to predict EGC lymph node metastasis with better accuracy and clinical performance.

## Introduction

Gastric cancer is the fourth leading cause of cancer-related deaths worldwide and the third leading cause of cancer-related deaths in China (Cao et al., 2021; Nagaraju et al., 2021; Sung et al., 2021; Varon et al., 2021). It is estimated that 478,508 new cases of gastric cancer are diagnosed in China each year (Cao et al., 2021). More than 80% of Chinese patients are diagnosed at an advanced stage, with a low 5-year survival rate of 44.09–59.0% (Ji et al., 2018; Cao et al., 2021).

Early gastric cancer (EGC) cases can be more easily detected with improvements in methods of early detection. EGC is defined as a tumor confined to the mucosa and/or submucosa, independent of the lymph node status (Japanese Gastric Cancer, 2011) and typically has a good prognosis (Sano et al., 2017). However, there have been reports on the risk of lymph node metastasis and treatment failure for EGC (Saragoni et al., 1998; Saragoni et al., 2000). Well-developed techniques in function-preserving gastrectomy have been used to improve the quality of life of patients with EGC, such as endoscopic submucosal dissection (ESD), local gastrectomy, segmental gastrectomy, and pylorus-preserving gastrectomy (Nomura and Okajima, 2016). ESD is a recently developed technique that is widely accepted for the treatment of EGC (Ono et al., 2021), with greater preservation of function, reduced postoperative complications/cost, and preserved quality of life than gastrectomy; meanwhile, ESD requires experienced and highly skilled endoscopists (Yada et al., 2013; Gotoda et al., 2014). Endoscopic surgery is used to dissect the mucosa or submucosa, and regional lymph nodes are not treated. Furthermore, local gastrectomy, segmental gastrectomy, and pylorus-preserving gastrectomy constitute investigational treatments and should be prospectively verified in randomized clinical trials (RCTs) (Japanese Gastric Cancer, 2021). Regarding the extremely strict indications for ESD (Ono et al., 2021), few patients with EGC can benefit from function-preserving gastrectomy. Additionally, the metachronous metastasis rate was significantly higher in an ESD group than that in a surgery group (Lee et al., 2018). Although standard radical surgery may yield survival benefits for a small number of patients, it may also introduce additional surgical risks to many patients without lymph node metastasis. Therefore, the development of an accurate predictive tool for assessing the risk of lymph node metastasis in EGC is urgently needed.

Nomogram is an intuitive tool for the individual probability of a clinical event based on a statistical predictive model (Iasonos et al., 2008) to quantify risk factors for lymph node metastasis in several human cancers (Briganti et al., 2012). To date, several studies have explored the independent high-risk factors for lymph node metastasis in EGC and established prediction models with good performance scores ranging from 0.813 to 0.860 (Zheng et al., 2016; Kim et al., 2020). Age, sex, ulceration, invasion depth, histology, differentiation, and lymphovascular invasion were considered high-risk factors and were included in different nomograms (Zheng et al., 2016; Mu et al., 2019; Kim et al., 2020; Sui et al., 2021). Although previous studies have established nomograms with good performance, all the variables involved were preoperatively unavailable.

Human epidermal growth factor receptor 2 (HER2)–positive gastric cancer is a unique disease subtype (Tolmachev et al., 2021). HER2 amplification or protein overexpression occurs in up to 20% of gastric cancer cases (Okines et al., 2012; Gordon et al., 2013). A previous study showed that HER2 is associated with poor prognosis in EGC without lymph node metastasis (Yan et al., 2015). Currently, there is no predictive nomogram that includes the HER2 status to determine the risk of lymph node metastasis in EGC, especially in East Asia, which has a high incidence of gastric cancer. In the Trastuzumab for Gastric Cancer (ToGA) trial, the overall HER2 positive rate was 23.2% for biopsy specimens and 19.7% for surgical specimens, which makes HER2 an available molecular phenotype recommended for preoperative evaluation (Van Cutsem et al., 2015). In the recommendation by the Chinese Society of Clinical Oncology (CSCO) Guidelines, Version 2021 (Wang et al., 2021), during the preoperative diagnostic process, the HER2 expression status needs to be examined and clarified. Thus, we attempted to establish nomogram models of lymph node metastasis in EGC before surgery and to determine whether they can accurately predict lymph node metastasis in patients with EGC *via* HER2 detection by analyzing the clinicopathological data used in the models.

## Materials and Methods

### Patients

**FIGURE 1:**
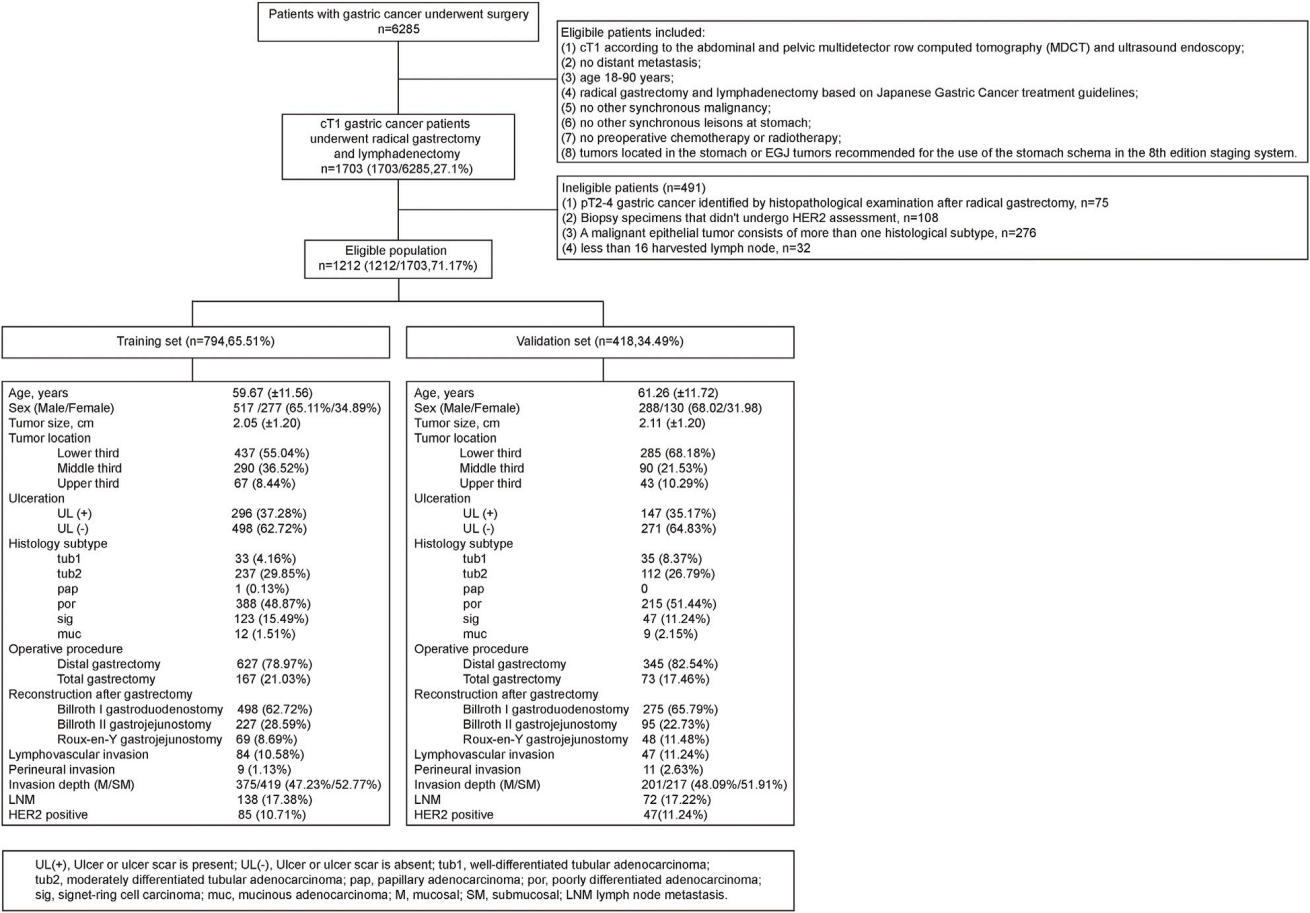
Flow diagram of patient enrollment and characteristics of patients in the training set and validation set.

This case-control study used data from a prospectively collected database at the Shanghai Jiao Tong University School of Medicine Affiliated Ruijin Hospital. From January 2012 to August 2018, a total of 6,285 patients with gastric cancer underwent surgery at Ruijin Hospital, Shanghai Jiao Tong University. The eligibility criteria are illustrated in the flow diagram (Figure 1). Standard gastrectomy is the principal surgical procedure performed with a curative intent. It involves resection of at least two-thirds of the stomach, with D2 lymphadenectomy for cT1N + tumors and D1/D1+ lymphadenectomy for cT1N0 tumors. Only patients who did not receive preoperative therapy were included in the study. Exclusion criteria were as follows: (i) pT2-4 gastric cancer identified by histopathological examination after radical gastrectomy; (ii) biopsy specimens that did not undergo HER2 assessment; (iii) a malignant epithelial gastric tumor consisting of more than one histological subtype (the different type of histological components was excluded in order to simplify the histological subtype factors); and (iv) less than 16 harvested lymph nodes (Figure 1).

Patients who underwent surgery between January 2012 and December 2016 were assigned to the training set, and patients who underwent surgery between January 2017 and August 2018 were enrolled in the validation set. The prediction model was developed in the training sets and tested in the validating sets. Ethical approval was obtained from the Ruijin Hospital Ethics Committee, Shanghai Jiao Tong University School of Medicine, China (No. 2018–151), and written informed consent was obtained from all patients. All procedures in this retrospective study were in compliance with the Helsinki Declaration.

### HER2 Evaluation

At the time of diagnosis, HER2 testing is recommended for all patients with gastric adenocarcinoma. The National Comprehensive Cancer Network (NCCN) guidelines recommend that immunohistochemistry (IHC) should be performed first followed by the Hofmann-modified scoring system (Hofmann et al., 2008; Rüschoff et al., 2010) for assessment of the HER2 status. An IHC score of 0 or 1 + indicates a negative result for HER2 expression, 2 + indicates an equivocal result that should be confirmed with *in situ* hybridization (ISH), and 3 + or positive amplification on ISH indicates a positive result for the HER2 expression. HER2 testing was performed at the Central Laboratory, Department of Pathology, Ruijin Hospital.

### Outcome and Covariates

Patients with pathologically diagnosed EGC were divided into two groups based on their postoperative pathological results, with or without lymph node metastasis.

The clinicopathological characteristics, including age, sex, tumor size, location, presence or absence of ulceration, invasion depth, histological subtype, HER2 status, lymphovascular invasion, and perineural invasion, were retrieved from medical records. Tumor size, presence or absence of ulceration, and location were obtained from endoscopic examinations. The histology type and HER2 status were determined using biopsy specimens. Invasion depth was determined using abdominal and pelvic multidetector-row computed tomography (MDCT) and ultrasound endoscopy. Lymphovascular invasion and perineural invasion were determined based on postoperative histopathological findings.

Lymph node metastasis was determined based on the indications for ESD recommended by the Japanese Gastric Cancer Association (JGCA). Based on the guidelines for ESD and endoscopic mucosal resection for EGC (second edition) (Ono et al., 2021), the absolute indications for endoscopic treatment were as follows: (i) differentiated intramucosal carcinoma with a maximum diameter of ≤2 cm and without ulcerative lesions; (ii) differentiated intramucosal carcinoma with a maximum diameter of >2 cm and without ulcerative lesions; (iii) cT1a with a diameter of ≤3 cm and ulceration [UL (+)]; and (iv) undifferentiated intramucosal carcinoma with a maximum diameter of ≤2 cm and without ulcerative lesions. The terminology used in this study is based on the Japanese classification of gastric carcinoma.

### Statistical Analysis

Categorical variables are summarized as frequencies and percentages. Continuous variables are summarized using medians and ranges. The training set was employed for risk factor identification and prediction model development. Within the training set, univariable and multivariable logistic regression analyses were performed to identify the clinical parameters associated with lymph node metastasis. Odds ratios (ORs), 95% confidence intervals (CIs), and *p*-values were reported. Clinical parameters significantly associated with lymph node metastasis in univariate analysis were included in the multivariate analysis, and a prediction model was developed. Model validation and nomogram construction were performed using a previously described method (Iasonos et al., 2008). A predictive nomogram for lymph node metastasis was built based on the prediction model.

The accuracy of the nomogram was evaluated based on the discrimination ability and the calibration plot in the training set. The receiver operating characteristic (ROC) curve was plotted, and the area under the ROC curve (AUC) with a 95% CI was calculated to quantify the discrimination ability of the nomogram. The AUC of 1.0 corresponds to the best model prediction, and the AUC of 0.5 represents a random prediction. Calibration curves were used to detect the consistency between actual lymph node metastasis and the predicted probability of lymph node metastasis using the nomogram. Moreover, a calibration plot was generated using 2000 repetitions of bootstrap sample corrections. The validation set was used to examine the external validity of the prediction model using the ROC plot.

Finally, using the decision curve analysis described by Vickers et al. (Vickers and Elkin, 2006), we assessed the clinical result achieved after using the prediction model for treatment selection by quantifying the net benefit at different threshold probabilities and comparing the net benefit with the absolute criteria for ESD. All analyses were performed using R version 3.4.3 (R-Foundation, Vienna, Austria), with two-sided *p*-values reported and significance considered at *p* < 0.05.

## Results

### Clinicopathological Features of Patients With EGC

In all, 1,212 patients were included in this analysis, including 805 men (66.23%) and 407 (33.77%) women. The median patient age was 61 years. The number of poorly differentiated tumors and moderately differentiated tumors was 794 (36.7%) and 418 (65.51%), respectively. Mucosal invasion was detected in 576 patients (47.52%), and submucosal invasion was detected in 636 patients (52.48%). Additionally, 132 cases (10.89%) were positive for the HER2 expression, and 210 patients (17.33%) had lymph node metastasis.

A total of 794 patients who underwent surgery before January 2017 were enrolled in the training set, and the remaining 418 patients were assigned to the validation set. There were no significant differences in the clinicopathological characteristics between the training and validation sets, with the exception that tumors in the validation set were more likely to occur in the lower part of the stomach.

### Univariate Analysis of Lymph Node Metastasis in the Training Set

In the univariate analysis, lymph node metastasis was significantly associated with age (*p* = 0.078), tumor size (*p* = 0.003), ulceration (*p* = 0.002), submucosal invasion (*p* < 0.001), histology subtype (*p* < 0.001), lymphovascular invasion (*p* < 0.001), perineural invasion (*p* = 0.007), and HER2 positivity (*p* < 0.001). Continuous variables were converted to binary variables, and the cut-off point of age was determined by maximizing the sum of sensitivity and specificity after spline smoothing, which was 55 years within the training set. Analysis showed that patients with EGC aged ≤ 55 years had a greater risk of lymph node metastasis. In terms of the histological subtype, poorly differentiated adenocarcinoma (Por, *p* < 0.001) was an independent risk factor for lymph node metastasis in EGC, while signet-ring cell carcinoma (Sig, *p* = 0.131) and mucinous adenocarcinoma (Muc, *p* = 0.311) did not present a higher risk of lymph node metastasis than well or moderately differentiated tubular adenocarcinomas (Table 1).

**TABLE 1:** Univariate analysis and multivariate analysis (Clinical nomogram model) of lymph node metastasis in early gastric cancer in the training set.

Clinicopathological	Univariate logistic regression		Multivariate logistic regression	
Parameters	OR (95% CI)	*P*	OR (95% CI)	*P*
Age as continuous variable, years	0.99 (0.97, 1.00)	0.078	—	
Age as categorical variable, years				
≤ 55	1.00	—	1.00	—
> 55	0.60 (0.42, 0.88)	0.009	0.56 (0.37, 0.86)	0.007
Sex			—	
Male	1.00			
Female	1.20 (0.82, 1.75)	0.340		
Size as continuous variable, cm	1.23 (1.07, 1.42)	0.003		
Size as categorical variable, cm				
≤ 2	1.00	—	1.00	—
2–3	1.75 (1.13, 2.68)	0.012	1.64 (1.03, 2.60)	0.037
>3	2.00 (1.17, 3.34)	0.009	2.04 (1.14, 3.57)	0.015
Tumor Location			—	
Lower third	1.00	—		
Middle third	0.80 (0.53, 1.18)	0.260		
Upper third	0.85 (0.41, 1.64)	0.645		
Ulceration				
UL (−)	1.00	—	1.00	—
UL (+)	1.82 (1.26, 2.64)	0.002	1.23 (0.81, 1.85)	0.326
Histology subtype				
tub1/tub2/pap	1.00	—	1.00	—
Sig	1.69 (0.84, 3.33)	0.131	2.27 (1.08, 4.75)	0.029
Muc	2.26 (0.33, 9.29)	0.311	1.81 (0.26, 8.00)	0.481
Por	3.82 (2.38, 6.40)	< 0.001	3.48 (2.08, 6.03)	< 0.001
Lymphovascular invasion	5.04 (3.11, 8.13)	< 0.001	—	
Perineural invasion	6.13 (1.60, 25.04)	0.007	—	
Submucosal invasion	4.20 (2.74, 6.60)	< 0.001	3.44 (2.16, 5.61)	< 0.001
HER2 positive	3.04 (1.84, 4.93)	< 0.001	2.66 (1.52, 4.62)	< 0.001

UL (−), ulcer or ulcer scar is absent; UL (+), ulcer or ulcer scar is present; tub1, well-differentiated tubular adenocarcinoma; tub2, moderately differentiated tubular adenocarcinoma; pap, papillary adenocarcinoma; sig, signet-ring cell carcinoma; muc, mucinous adenocarcinoma; por, poorly differentiated adenocarcinoma.

### Preoperative Nomogram (Clinical Model) of Lymph Node Metastasis in EGC

A preoperative predictive nomogram containing important factors related to EGC lymph node metastasis was constructed based on the logistic regression model. In order to compare the ESD indications, all parameters consisting of ESD indications and clinicopathological risk factors of lymph node metastasis of EGC in the univariate analysis were included in the multivariable analysis.

Multivariate logistic regression analysis showed that age >55 years (OR: 0.56, 95% CI: 0.37–0.86, *p* = 0.007), tumor size of 2–3 cm (OR: 1.64, 95% CI: 1.03–2.60, *p* = 0.037), tumor size >3 cm (OR: 2.04, 95% CI: 1.14–3.57, *p* = 0.015), submucosal invasion (OR: 3.44, 95% CI: 2.16–5.61, *p* < 0.001), histological subtype of Sig (OR: 2.27, 95% CI: 1.08–4.75, *p* = 0.029), histological subtype of Por (OR: 3.48, 95% CI: 2.08–6.03, *p* < 0.001), and HER2 positivity (OR: 2.66, 95% CI: 1.52–4.62, *p* < 0.001) were independent risk factors for lymph node metastasis in EGC (Table 1).

**FIGURE 2:**
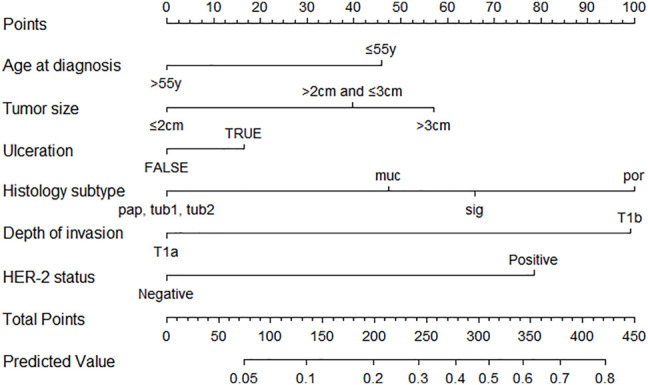
Nomogram for predicting lymph node metastasis in EGC patients. EGC, early gastric cancer; pap, papillary adenocarcinoma; tub1, well-differentiated tubular adenocarcinoma; tub2, moderately differentiated tubular adenocarcinoma; sig, signet-ring cell carcinoma; muc, mucinous adenocarcinoma; por, poorly differentiated adenocarcinoma.

**FIGURE 3:**
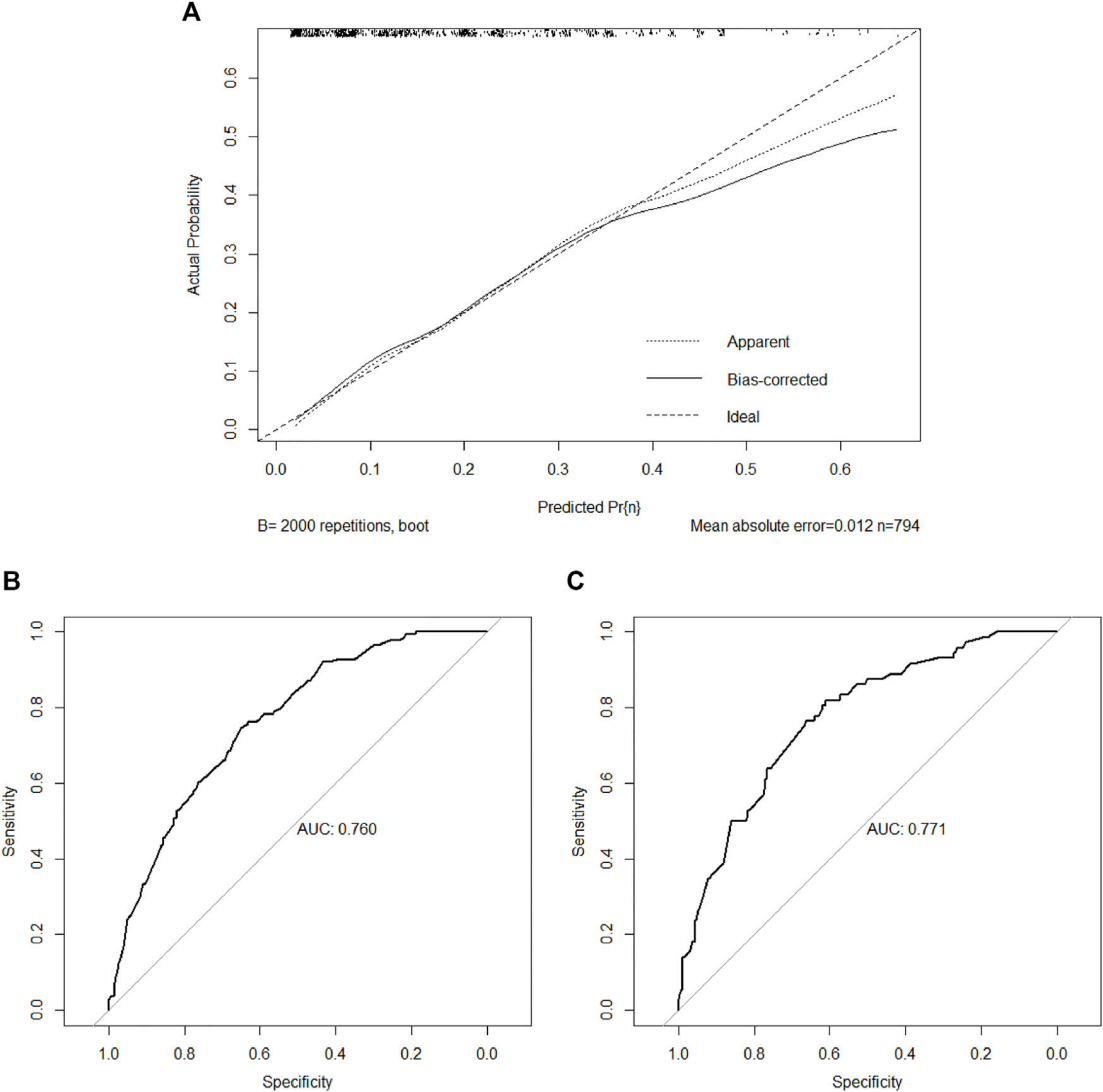
Assessment of the nomogram for predicting lymph node metastasis in the training set and validation set. **(A)** Calibration plot in the training set. After 2000 repetitions, the bootstrap-corrected calibration curve (solid line) lay close to the ideal reference line (dashed line), which demonstrated a perfect agreement between the predicted and actual outcomes (mean absolute error = 0.012); **(B)** ROC plot in the training set. The AUC of the ROC was 0.760 (95% CI, 0.719–0.800); **(C)** ROC plot in the validation set. The AUC of the ROC was 0.771 (95% CI, 0.714–0.828). ROC: receiver-operating characteristic; AUC: area under the ROC curve.

The nomogram revealed that the histological subtype had the greatest impact on scoring, followed by invasion depth and HER2 status. The effects of tumor size and age on the model performance were not significant. Each level in the variable was summed by the total score based on the point scale and positioned on the total score scale to determine the corresponding lymph node metastasis probability of each patient (Figure 2). With an additional 2000 bootstraps, the correction diagram showed good consistency between the deviation correction prediction and the ideal reference line (mean absolute error = 0.012, Figure 3A). The Hosmer–Lemeshow test yielded a *p* value of 0.677, indicating that this model was suitable as a prediction model. After 2000 bootstrap repetitions, the AUC of the internal validation in the training set was 0.760 (95% CI = 0.719–0.800, Figure 3B). The AUC of external validation in the validation set was 0.771 (95% CI = 0.714–0.828, Figure 3C), indicating the good performance of this nomogram.

### Effect of the HER2 Expression on Lymph Node Metastasis in EGC

Univariate analysis showed significant differences in the size of EGC lesions (*p* = 0.012), presence or absence of ulceration (*p* = 0.002), depth of invasion (*p* < 0.001), tumor location (*p* < 0.001), histological subtype (*p* < 0.001), and lymphovascular invasion (*p* < 0.001) between the two groups with different HER2 statuses. Subsequently, the multivariate stepwise analysis confirmed that tumors located in the upper third of the stomach (OR: 2.41, 95% CI: 1.42–4.07, *p =* 0.001), submucosal invasion (OR: 0.40, 95% CI: 0.25–0.63, *p* < 0.001), histology subtype of tub1/tub2/pap (OR: 1.75, 95% CI: 1.14–2.68, *p* = 0.010), and lymphovascular invasion (OR: 0.37, 95% CI: 0.22–0.60, *p* < 0.001) were independent risk factors of HER2 positivity in patients with EGC (Table 2).

**TABLE 2:** Clinicopathological factors associated with the HER2 expression in 1,212 early gastric cancer cases.

Clinicopathological parameters	Univariate analysis		*P* (Chi-square)	Multivariate analysis OR (95%CI)^a^, *P*
	HER2 positive (*n* = 132)	HER2 negative (*n* = 1,080)		
Age (years)			0.181 (5.59)	
≤ 55	30 (22.73%)	360 (33.33%)		1.34 (0.86, 2.09), 0.201
>55	102 (77.27%)	720 (66.67%)		
Sex			0.721 (0.127)	
Male	90 (68.18%)	715 (66.2%)		1.05 (0.69, 1.58), 0.826
Female	42 (31.82%)	365 (33.8%)		
Ulceration			0.002 (9.683)	
UL (+)	65 (49.24%)	378 (35%)		0.70 (0.47, 1.03), 0.067
UL (-)	67 (50.76%)	702 (65%)		
Tumor size (cm)			0.012 (8.88)	
≤ 2	80 (60.6%)	726 (67.22%)		1.54 (0.92, 2.56), 0.098
2–3	26 (19.7%)	237 (21.94%)		
>3	26 (19.7%)	117 (10.83%)		
Invasion Depth			< 0.001 (31.159)	
M	32 (24.24%)	544 (50.37%)		0.40 (0.25, 0.63), < 0.001
SM	100 (75.76%)	536 (49.63%)		
Tumor location			< 0.001 (23.564)	
Upper third	27 (20.45%)	83 (7.69%)		2.41 (1.42, 4.07), 0.001
Middle third	39 (29.55%)	341 (31.57%)		
Lower third	66 (50%)	656 (60.74%)		
Histology subtype			< 0.001 (18.623)	
tub1/tub2/pap	62 (47%)	356 (32.96%)		1.75 (1.14, 2.68), 0.010
Sig	5 (3.79%)	165 (15.28%)		
Muc	1 (0.76%)	20 (1.85%)		
Por	64 (48.48%)	539 (49.91%)		
LVI			< 0.001 (32.62)	
Present	34 (25.76%)	97 (8.98%)		0.37 (0.22, 0.60), < 0.001
Absent	98 (74.24%)	983 (91.02%)		
PNI			1.00000^a^	
Present	2 (1.52%)	18 (1.67%)		2.91 (0.63, 13.42), 0.172
Absent	130 (98.48%)	1,062 (98.33%)		

a Comparisons between enumeration data were conducted by the fisher exact method.

UL (+), ulcer or ulcer scar is present; UL (−), ulcer or ulcer scar is absent; M, mucosal; SM, submucosal; tub1, well-differentiated tubular adenocarcinoma; tub2, moderately differentiated tubular adenocarcinoma; pap, papillary adenocarcinoma; sig, signet-ring cell carcinoma; muc, mucinous adenocarcinoma; por, poorly differentiated adenocarcinoma; LVI, lymphovascular invasion; PNI, perineural invasion.

The intergroup analysis showed that there were 145 patients with EGC who satisfied the first absolute ESD indication, and two (1.38%) of these patients had lymph node metastasis. Among the two patients, one had a positive HER2 status, and the positivity rate was 50% (Table 3). However, four (5.63%) patients with EGC who completely satisfied the second absolute ESD indication had lymph node metastasis, while none of the four patients had a positive HER2 status (Table 3). Additionally, the intergroup analysis revealed that 10.71% of patients with EGC had lymph node metastasis when selecting patients who satisfied the third absolute ESD indication (Table 3). One of these six patients had a positive HER2 status, with a positivity rate of 16.67%. Among the 109 patients with EGC who satisfied the fourth absolute ESD indication, 16 (14.68%) had lymph node metastasis. Two of 16 patients had a positive HER2 status, with a positivity rate of 12.5%.

**TABLE 3:** Intergroup analysis between HER2 positive and lymph node metastasis (LNM) in ESD indication for EGC according to the JGCA guidelines.

ESD indications	LMN		LMN with HER2 positive		Proportion in LNM group with HER2 positive
	Yes	No	Yes	No	
(1)	2 (1.38%)	143 (98.62%)	1	1	1/2 (50%)
(2)	4 (5.63%)	67 (94.37%)	0	4	0/4 (0%)
(3)	6 (10.71%)	50 (89.29%)	1	5	1/6 (16.67%)
(4)	16 (14.68%)	93 (85.32%)	2	14	2/16 (12.5%)

1 Differentiated-type adenocarcinoma without ulcerative findings (UL (−)), of which the depth of invasion is clinically diagnosed as T1a and the diameter is ≤2 cm.

2 Tumors clinically diagnosed as T1a and of differentiated-type, UL (−), but >2 cm in diameter.

3 Tumors clinically diagnosed as T1a and of differentiated-type, UL (+), and ≤ 3 cm in diameter.

4 Tumors clinically diagnosed as T1a and of undifferentiated-type, UL (−), but ≤ 2 cm in diameter.

### Clinical Value of ESD Indications and Nomogram

**FIGURE 4:**
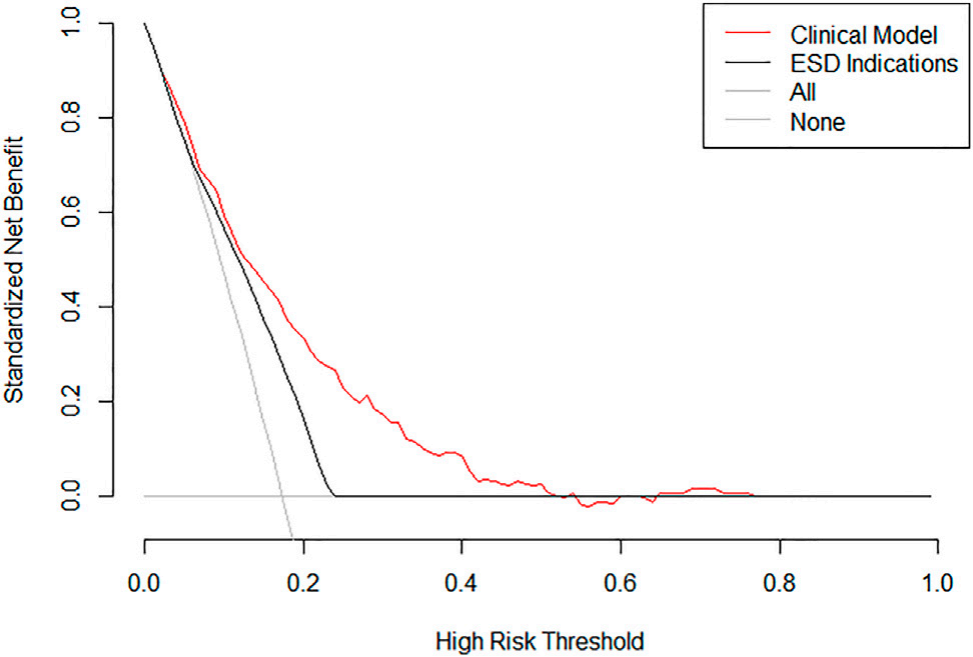
Clinical performance of the clinical model (nomogram) and ESD indications. Decision curve analysis on the clinical model (nomogram) (red line) and ESD absolute indications recommended by JGCA (solid line). The *y*-axis represents net benefits, calculated by subtracting the relative harms (false positives) from the benefits (true positives). The *x*-axis measures the threshold probability.

The clinical performance of the JGCA absolute indications for ESD and the clinical model (nomogram) are shown in Figure 4 and Table 4. Due to the high survival rate after surgical resection, missed cancer diagnosis, rather than over-diagnosis, would have unacceptable consequences. Therefore, decision curve analysis was used to determine the relative value between false negative and false positive errors (termed net benefit). Compared with the two simple strategies of performing radical gastrectomy and lymphadenectomy for all patients (sloping solid gray line) or no patients (horizontal solid gray line), the clinical model (nomogram) had a greater value in predicting the development of treatment strategies than the absolute indications of ESD and exhibited an excellent net benefit over the range of threshold probabilities. For example, the value of net benefits would be 0.103 if we selected 10% as the cut-off value, indicating that the clinical model (nomogram) would identify approximately 10 patients with lymph node metastasis among 100 patients compared with simple observation, without adding any unnecessary resections (false positives).

**TABLE 4:** Clinical performances between the nomogram and ESD indication.

Threshold probability (%)	Net benefits per 100 patients			Nomogram			
	Treat all	Nomogram	ESD indication	Sensitivity (%)	Specificity (%)	FNR (%)	NPV (%)
5	13.0	13.9	13.6	99.3	22.9	0.7	99.3
10	8.2	10.3	9.4	88.4	44.5	11.6	94.8
15	2.8	7.88	4.6	76.8	62.5	23.2	92.8
20	−3.3	5.64	−0.7	63.8	73.6	36.2	90.6

ESD, endoscopic submucosal dissection; FNR, false negative rate; NPV, negative predictive value.

## Discussion

### Main Findings

Neoplasms remain the main cause of death worldwide (Palle et al., 2020; Navashenaq et al., 2021; Zhang et al., 2021). In this study, we found that with integration of the HER2 status, a clinical prediction model and an effective nomogram could predict EGC lymph node metastasis with better accuracy and clinical performance.

### Interpretation

ESD has proven to be a safe and effective treatment when it meets the guideline indications for patients with EGC (Yamaguchi et al., 2009; Toyonaga et al., 2013). In addition to advances in treatment techniques, progress in the field of endoscopic devices and techniques now enables ESD for overall pathologic diagnosis (Fujimoto et al., 2017).

We performed ESD for patients with EGC until the preoperative diagnosis of lymph node metastasis was confirmed since lymph node invasion was difficult to assess even with improved techniques for imaging evaluations. On the other hand, since ESD indications are too broad for accurate predictions and the accuracy in estimating lymph node metastasis appears to be limited, few patients with EGC may benefit from ESD. As this study demonstrated, there were only 381 (31.44%) patients with EGC who completely met the absolute ESD indications, while 1,002 (82.67%) patients failed to present lymph node metastasis in our study. In addition, 28 of 381 patients who completely met the absolute ESD indications had lymph node metastasis at a rate of 7.3%, which was higher than the 1% possibility required for absolute indications for ESD (Japanese Gastric Cancer, 2021).

Quantitative predictive models benefit clinicians and patients in making more objective decisions regarding treatment options. To date, the predictive probability has not been clearly defined. The optimal threshold depends on the extent to which the patient or clinician rejects the risk. Fujikawa et al. (Fujikawa et al., 2015) reported that two-thirds of patients with clinical T1 gastric cancer are possible candidates for endoscopic treatment since the false-negative rate is 5%. In biopsies of breast cancer sentinel lymph nodes, the recognized false-negative rate is 5% (Qiu et al., 2016). Unlike ultrasound-guided biopsy of axillary lymph nodes in breast cancer, clinical diagnosis of gastric lymph node metastasis in EGC is difficult.

The incidence of lymph node metastasis in our study was 17.33%, which is similar to previous studies (Pereira et al., 2018; Yin et al., 2020; Mei et al., 2021). Although our study confirmed that lymphovascular invasion and perineural invasion were risk factors for lymph node metastasis in patients with EGC, clinicians could not obtain evidence of lymphovascular invasion and perineural invasion in the period of preoperative evaluation; thus, it was not included in the multivariate model. In the multivariate analysis, age, tumor size, histology, depth of invasion, and HER2 status were independent risk factors for lymph node metastasis. Li et al. (Li et al., 2018) found that male sex, age, depressed type, submucosal invasion, lymphovascular invasion, and tumor location were independent risk factors for lymph node metastasis in EGC. Oh et al. (Oh et al., 2021) demonstrated that in patients with EGC without lymphovascular invasion, tumor size >3 cm, submucosal invasion, and undifferentiated histologic type were significant risk factors for lymph node metastasis.

Previous studies have established nomograms to predict lymph node metastasis in EGC and have demonstrated a high-performance score (Zheng et al., 2016; Mu et al., 2019; Kim et al., 2020; Sui et al., 2021). However, previously established nomograms included lymphovascular invasion. Lymphovascular invasion has been suggested as an indicator of lymph node metastasis (Choi et al., 2021). However, lymphovascular invasion can only be obtained after endoscopy or gastrectomy. Thus, we aimed to establish a model using preoperative factors to better direct our selection of treatment methods.

In our study, the analysis revealed that among the 381 patients with EGC who fully met the absolute indications for ESD, 28 patients had lymph node metastasis and 4 (14.3%) had a positive HER2 expression. Studies have shown that the overexpression of HER2 is associated with invasive biological behavior and poor prognosis (Zhang et al., 2009; Lei et al., 2017). Han et al. (Han et al., 2020) found that the HER2 overexpression was significantly correlated with lymphovascular invasion and the presence of lymph node metastasis, which is consistent with our results.

Given that HER2 is directly related to lymphovascular invasion and lymph node metastasis, the detection of the HER2 expression is recommended for pathological evaluation in biopsy. Therefore, the inclusion of HER2 detection in preoperative evaluation can help clinicians make judgments and treatment decisions.

Based on the absolute indications for ESD and our results, we chose variables to predict the risk of lymph node metastasis for our nomogram in patients with EGC and to avoid unnecessary gastrectomy, which included the following: age, tumor size, ulceration, histology, depth of invasion, and HER2 status. Age, tumor size, ulceration, histology, depth of invasion, and HER2 status were easily obtained by routine preoperative examinations. The tumor size and the presence or absence of ulceration were obtained from endoscopic examination. Histology and HER2 status were determined using biopsy specimens. Invasion depth was determined using abdominal and pelvic multidetector-row computed tomography (MDCT) and ultrasound endoscopy. All these variables were easy to obtain; therefore, our nomogram had good application in clinical practice.

In our nomogram, the histological subtype of Por was dominant, and it was assigned 100 points; meanwhile, submucosal invasion was assigned 99 points, and HER2 positivity was assigned 78 points. Sizes over 3 cm, age ≤ 55 years, and ulceration were assigned relatively low points of 57, 46, and 17, respectively. The possibility of lymph node metastasis gradually increased with point accumulation. Our nomogram could predict the possibility of LNM for every individual patient, which may help clinicians make informed and customized decisions in clinical treatment. We demonstrated that our nomogram has good discrimination in both the training (AUC, 0.760) and validation sets (AUC, 0.771). In addition, clinical manifestation in the nomogram was superior to the absolute indications of ESD; therefore, its use may lead to the screening of more patients with EGC, with a negligible risk owing to excessive surgical resection.

### Limitations

To the best of our knowledge, this is the first study to provide a nomogram to predict the incidence of lymph node metastasis in EGC *via* the detection of the HER2 expression. Nevertheless, this study has some limitations. This was a single-center, retrospective study. Although we enrolled 794 patients in the training set and 418 in the validation set to validate the model internally and externally in independent cohorts, further external validation from other centers is needed. Additionally, this study was based on an Asian population with EGC. Thus, the results may not be extrapolated to other patient populations without further validation in an independent cohort. Moreover, we did not develop a specific cut-off value for lymph node metastasis for different treatments in patients with EGC. The cut-off value depended on how the patients and doctors ignored the risk. Therefore, compared with stratification, our nomogram is useful in providing patients and doctors with evidence to aid clinical decision-making. Despite these limitations, our nomogram served as an effective tool for predicting the incidence of lymph node metastasis in Chinese patients with EGC, which may lead to improved selection of appropriate treatments.

## Conclusion

In conclusion, we constructed a nomogram to predict the probability of lymph node metastasis in patients with EGC *via* HER2 detection. Our nomogram can be used not only for preoperative evaluation to determine whether standard radical gastrectomy is needed in patients with EGC at a high risk of lymph node metastasis but also for intraoperative evaluation to determine whether radical lymphadenectomy is necessary. The clinical performance of our nomogram is superior to that of the absolute indications of ESD in patients with EGC. Randomized clinical trials are needed to determine appropriate indications for function-preserving gastrectomy, which is still regarded as investigational treatment.

## Data Availability

The original contributions presented in the study are included in the article/Supplementary Material; further inquiries can be directed to the corresponding authors.
